# Nucleolin (NCL) inhibits the growth of peste des petits ruminants virus

**DOI:** 10.1099/jgv.0.001358

**Published:** 2019-12-03

**Authors:** Dandan Dong, Shiqiang Zhu, Qiuhong Miao, Jie Zhu, Aoxing Tang, Ruibin Qi, Teng Liu, Dongdong Yin, Guangqing Liu

**Affiliations:** ^1^​ Innovation Team of Small Animal Infectious Disease, Shanghai Veterinary Research Institute, Chinese Academy of Agricultural Sciences, Shanghai, 200241, PR China

**Keywords:** peste des petits ruminants virus, N protein, nucleolin

## Abstract

Peste des petits ruminants (PPR) is a highly contagious disease of small ruminants that is caused by peste des petits ruminants virus (PPRV). To date, the molecular mechanism of PPRV infection is still unclear. It is well known that host proteins might be involved in the pathogenesis process for many viruses. In this study, we first proved that nucleolin (NCL), a highly conserved host factor, interacts with the core domain of PPRV N protein through its C terminus and co-locates with the N protein in the nucleus of cells. To investigate the role of NCL in PPRV infection, the expression level of NCL was inhibited with small interfering RNAs of NCL, and the results showed that PPRV growth was improved. However, the proliferation of PPRV was inhibited when the expression level of NCL was improved. Further analysis indicated that the inhibitory effect of NCL on the PPRV was caused by stimulating the interferon (IFN) pathways in host cells. In summary, our results will help us to understand the mechanism of PPRV infection.

## Introduction

Peste des petits ruminants virus (PPRV) is the causative agent of peste des petits ruminants (PPR), a contagious disease of wild and domestic cloven-hoofed small ruminants that is responsible for significant financial losses worldwide [1]. PPRV belongs to the genus *Morbillivirus* within the family *Paramyxoviridae*, and its particles are enveloped and pleomorphic with a diameter of 400–500 nm [2]. The genome of PPRV is a single-stranded, negative-sense RNA molecule composed of 15 948 nucleotides [3]. It contains six transcriptional units that encode six structural (N, P, M, F, H and L) and two nonstructural (V and C) proteins in the order 3′–N–P(V/C)–M–F–H–L–5′ [4]. Among of the structural proteins, N protein is the most abundant and immunogenic protein, and thus N protein is widely used in diagnostic tests, disregarding the fact that it does not induce antiviral protective immunity [5–7]. Furthermore, it has been proved that N protein is essential for virus-like particle formation and viral replication of PPRV. Although morbilliviruses replicate in the cytoplasm, the nucleocapsid-like aggregates formed by N protein can be seen in both the cytoplasm and nucleus of mammalian transfected cells [8–12].

Although the precise role of N protein in the viral life cycle of PPRV remains unknown, some studies about the N protein of paramyxoviruses provide us with some clues to help unravel the possible roles of PPRV N protein. For example, N protein might affect the viral life cycle in multiple steps. It combines with M protein to facilitate virus assembly [13, 14] and is involved with P–L polymerase complex during replication and transcription [15, 16]. In addition, studies have shown that several host regulatory proteins, such as interferon (IFN) regulatory factor (IRF)3, heat shock protein (Hsp)72 and cell surface receptor interact with N protein and then affect the pathogenic process of morbillivirus [17, 18]. However, few host cell factors that interact with PPRV N protein have been identified, and the effect of the interaction between cytokines and N protein on PPRV replication is also less well known, which might be very important for understanding the pathogensis of PPRV.

In this study, we identified for the first time an important host cytokine (nucleolin, NCL) that interacts with PPRV N protein. NCL, formerly known as C23 [19], is an important multifunctional protein in eukaryotic cells. It is involved in the processing of chromatin organization and stabilization, DNA and RNA metabolism, the assembly of ribonucleoprotein complexes and nucleocytoplasmic transport [20–22]. The NCL contains three domains: the N-terminal domain or acid domain, the central domain or RNA recognition motifs (RRMs), and the C-terminal domain (CTD) or glycine/arginine-rich domain (GAR/RGG) [20]. The N-terminal domain (NTD) with a nuclear localization signal participates in many protein–protein interactions [23]; the central domain is involved in the processing and modification of pre-rRNA [24]; and the CTD interacts with a number of proteins and contributes to the interaction of NCL RNA binding domain(RBD) with targets [25–29].

Since NCL is widely distributed in nucleoli, the cytoplasm and the cell membrane, it plays an important role in the replication and intracellular transport of many viruses. For example, NCL is involved in the internalization of rabbit haemorrhagic disease virus through clathrin-dependent endocytosis [30]. NCL is necessary for various influenza A viruses, including H1N1, H3N2, H5N1 and H7N9, to enter the cells [31–34]. NCL could also mediate enterovirus (EV)71 binding to and infection of cells [35]. Contradictory to these conclusions, some studies have shown that NCL plays a negative regulatory role in the growth of some viruses. For instance, NCL can suppress hepatitis C virus replication by binding to and stabilizing viral core RNA G4 structure [36]. Likewise, NCL silences human immunodeficiency virus (HIV)-1 transcription by stabilizing G-quadruplex structures folded by the long terminal repeat promoter [37]. Furthermore, NCL has been proved to act as a novel potential antiviral factor during H5N1 infection [38].

To explore the role of NCL in PPRV growth, we first examined the interaction between NCL and PPRV N protein, and then discussed the effect of NCL on PPRV growth. Further, we have also tried to discover the mechanism through which NCL affects PPRV infection. Our results showed that NCL inhibits PPRV growth by stimulating the secretion level of IFN-β. Our study will be helpful for us to understand the pathogenic mechanism of PPRV.

## Methods

### Cells, virus and virus titre assays

Vero-SLAM and HEK293T cells were grown in Dulbecco’s modified Eagle’s medium (DMEM; Gibco) containing 10 % foetal bovine serum (FBS; Gibco). Endometrial epithelial cells (EECs) were grown in DMEM/F12 containing 10 % FBS (Gibco). All cells were cultured at 37 °C with 5 % CO_2_. PPRV Nigeria/75/1 vaccine strain was propagated in Vero-SLAM cells. The virus titres in the culture of PPRV-infected Vero-SLAM cells were determined by using the Reed–Muench method. Viral titres were expressed as 50 % tissue culture infective dose (TCID_50_) 0.1 ml^−1^.

### Plasmids

The PPRV N protein cDNA (GenBank accession no. HQ197753.1) was cloned into the mammalian expression vector p3 ×Flag-CMV10 vector (Sigma) with the *EcoRI* and *KpnI* restriction enzymes to generate the p3 ×Flag-N plasmid. The pHA-NCL plasmid encoding the NCL protein with a HA tag was obtained by cloning NCL cDNA (GenBank accession no.XM_012163051.2) into a pCMV-HA vector (Clontech). For prokaryotic expression of the glutathione S-transferase (GST)-tagged N protein, DNA encoding the N protein was subcloned into the pGEX-4T-1 vector (Invitrogen) with the *EcoRI* and *NotI* restriction endonucleases. Truncated mutants of NCL and PPRV N were generated from pHA-NCL and p3 ×Flag-N by conventional PCR (the primers will be made available upon request). All constructs were confirmed by sequencing (Sangon Biotech).

### Plasmids DNA transfection

Vero-SLAM, HEK293T and EECs in six-well plates (Corning) were transfected with specific plasmids (3 µg each) with Lipofectamine 2000 (Invitrogen) according to the manufacturer’s instructions. At 6 h post-transfection, DMEM was supplemented with 1 % FBS instead of transfection medium and allowed to continue to culture for 48 h before being used for assays.

### Virus infection and treatment

Twenty-four hours after the transfection of DNA or siRNA, cells were infected with the PPRV Nigeria/75/1 vaccine strain at a multiplicity of infection (m.o.i.) of 3. After 2.5 h, the viral inoculum was discarded and the infected cells were washed twice with ice-cold PBS (pH 7.4) and cultured in DMEM supplemented with 1 % serum. Cell samples were collected at specific times after infection and stored at −80 °C until use.

### GST pulldown assays

GST-N and GST protein were expressed in competent *Escherichia coli* BL21 (DE3) cells, which were seeded in 1 ml overnight starter culture and then grown at 220 r.p.m. and 37 °C in 100 ml lysate until the mid-log phase (OD_600_=0.6–0.8). The cells were then induced by 0.2 mM isopropyl β-D-1-thiogalactopyranoside (IPTG) and cultured at 16 °C and 220 r.p.m. for 17 h. After the cells had been centrifuged at 5000 ***g*** for 15 min, the supernatant was discarded and the precipitate was stored at −80 °C. The precipitate was resuspended in lysis buffer (20 mM Tris/HCl pH 7.4, 60 mM NaCl, 1 mM ethylenediaminetetraacetic acid, 1 mg ml^−1^ lysozyme, 1 mM dithiothreitol and 0.1 % Triton X-100) supplemented with protease inhibitor for 1 h on ice. The lysates were centrifuged at 4 °C and 12 000 ***g*** for 15 min.

For the GST pulldown assays, GST-N and GST protein expressed in *E* *.* *coli* BL21 (DE3) cells were conjugated to glutathione/sepharose beads (Thermo Fisher Scientific) for 2 h at 4 °C. After being washed with wash buffer, the beads were incubated for overnight at 4 °C with HA-tagged NCL harvested from transfected HEK293T cells. After at least six washes with wash buffer, the bound proteins of the beads were identified by SDS-PAGE and immunoblotting.

### Co-IP

HEK293T cells were transfected with specific constructs by using Lipofectamine 2000 (Invitrogen). At 48 h after transfection, the cells were washed twice with cold PBS and lysed with co-IP lysis buffer (0.025 M Tris, 0.15 M NaCl, 0.001 M EDTA, 1 % NP-40, 5 % glycerol, pH 7.4) for 10 min on ice. The clarified extracts, pretreated with contrast resin, were incubated with resins plus anti-Flag monoclonal antibody (MAb) or anti-HA monoclonal antibody (MAb) overnight at 4 °C. After being washed five times with the wash buffer, the eluted proteins were boiled in 5× SDS sample buffer and separated by SDS-PAGE, followed by immunoblotting analysis with specific antibodies.

### Real-time qRT-PCR

To quantify the level of PPRV genomic copies, total RNA was extracted from PPRV-infected cells with TRIzol (Invitrogen) and treated with DNase I. The RNA was then reverse-transcribed to cDNA with Moloney murine leukaemia virus reverse transcriptase and random primers (Promega) according to the manufacturer’s instructions. All samples were analysed by qRT-PCR using the SYBR PreMix Ex *Taq* II kit (Takara). The relative abundance of target mRNA was normalized using endogenous glyceraldehyde 3-phosphate dehydrogenase (GADPH).

### RNA interference

siRNAs targeting NCL were synthesized at Sigma. The siRNAs were transfected into Vero-SLAM cells with Lipofectamine 2000 (Invitrogen). At 24 h post-transfection, cell were infected with PPRV as described above and collected at a specific time for qRT-PCR, Western blotting and virus titre assays.

### Western blotting

Protein samples were separated by 10–15% gels and transferred to nitrocellulose membranes (GE Healthcare) using a semi-dry transfer cell (Bio-Rad Laboratories). Membranes were blocked with 5 % nonfat milk in TBS–Tween (TBST) buffer (150 mM NaCl, 20 mM Tris and 0.05 % Tween-20, pH7.3) for 2 h at room temperature and then incubated with the following antibodies: anti-HA mouse MAb (1 : 1000; Abcam); anti-Flag mouse MAb (1 : 1000; Sigma); anti-N mouse MAb (1 : 2000; produced in-house); anti-NCL mouse MAb (1 : 500; Santa Cruz Biotechnology); anti-GST mouse MAb (1 : 2000; CW); and anti-actin mouse MAb (1 : 2000; CW). After being washed five times for 5 min each time, the membranes were incubated with a goat anti-mouse secondary antibody conjugated to horseradish peroxidase (1 : 10000; Sigma) for 1 h at room temperature. Blots were analysed by enhanced chemiluminescence (Thermo Fisher Scientific) using an automatic chemiluminescence imager (Tanon).

### Immunofluorescence assay (IFA)

HEK 293 T cells grown in 12-well plates (Corning) were cotransfected with pHA-NCL and p3 ×Flag-N. After 36 h transfection, cells were fixed with 4 % paraformaldehyde in PBS (Biosharp) for 30 min and permeabilized by 0.1 % Triton X-100 for 15 min. The cells were blocked with 5 % nonfat milk in PBS overnight at 4 °C and subsequently incubated with primary antibodies (mouse anti-HA MAb, 1 : 1000; rabbit anti-Flag MAb, 1 : 1000) for 1.5 h at 37 °C. After being washed five times for 5 min each time, the cells were incubated with secondary antibodies [Alexa Fluor 488 goat anti-rabbit IgG (H+L), 1 : 2000; Alexa Fluor 594 goat anti-mouse IgG (H+L), 1 : 2000] for 1 h. The cells were stained with 4′,6-diamidino-2-phenylindole (DAPI, Thermo Fisher Scientific) for 15 min and observed using the Zeiss SP2 confocal system (Leica Microsystems, Germany).

### Dual-luciferase (Luc) reporter assay

HEK 293 T cells grown in 12-well plates (Corning, USA) were transfected with 1 µg specific plasmid and additionally 200 ng pIFN-β-Luc or PRDII, 40 ng pRL-TK (Promega, USA) as an internal control. Reporter gene activity was analysed using the Dual-Luciferase Reporter 1000 assay system (Promega, USA) and measured with a TD-20/20 luminometer (Turner Designs, USA) according to the manufacturer’s instructions. Three independent experiments were performed in duplicate.

### Statistical analysis

Data were analysed statistically with Student’s *t*-test using SAS 9.1 software. *P*<0.05 was considered statistically significant.

## Results

### NCL specifically binds to PPRV N protein in infected cells

**Fig. 1.**
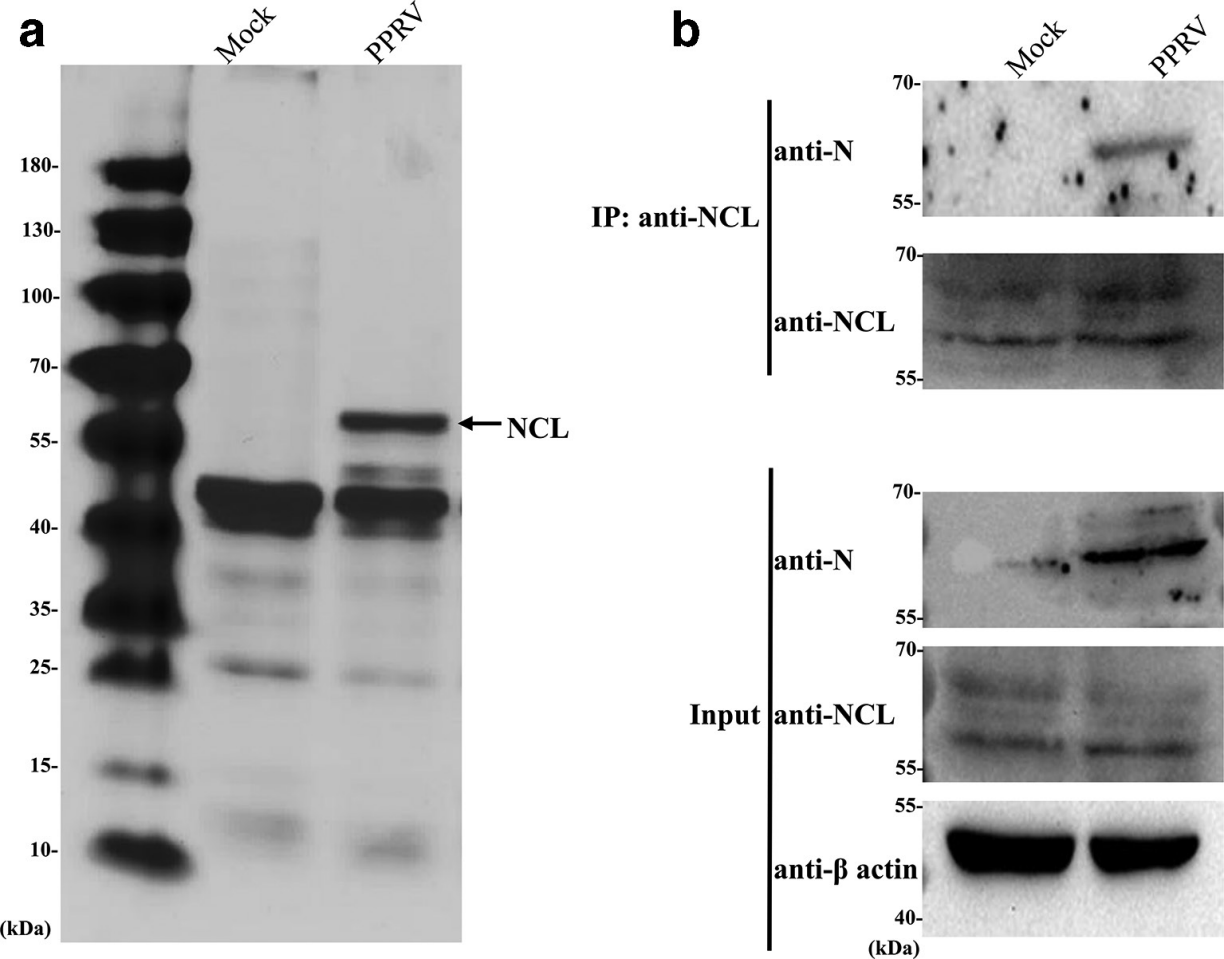
Identification of PPRV N protein-interacting protein by IP. (a) Vero-SLAM cells infected with PPRV or mock-infected with PBS were used for IP. The eluted proteins were isolated by 12 % SDS-PAGE, and then Coomassie staining and MS analysis were performed. (b) NCL interacted with PPRV N during natural viral infection. Vero-SLAM cells in 10 cm^2^ dishes were mock-infected with PBS or infected with PPRV at an m.o.i. of 3. After 72 h, cell samples were immunoprecipitated with mouse anti-NCL MAb and separated by 12 % SDS-PAGE, followed by Western blotting with mouse anti-N and mouse anti-NCL MAb, respectively.

To screen host cytokines interacting with PPRV N protein, a resin that binds to PPRV N monoclonal antibodies (MAb) was used as a bait, and Vero-SLAM cells infected with PPRV or cells mock-infected with PBS were preyed. The eluted proteins were subjected to mass spectrometry (MS). As shown in Fig. 1a, NCL was identified as an N protein-binding protein. Similarly, cell lysates infected with PPRV were immunoprecipitated with an anti-NCL MAb, followed by Western blotting for the detection of N protein with anti-N MAb. N protein was detected in PPRV-infected cells (Fig. 1b), indicating that N protein specifically interacted with NCL.

**Fig. 2.**
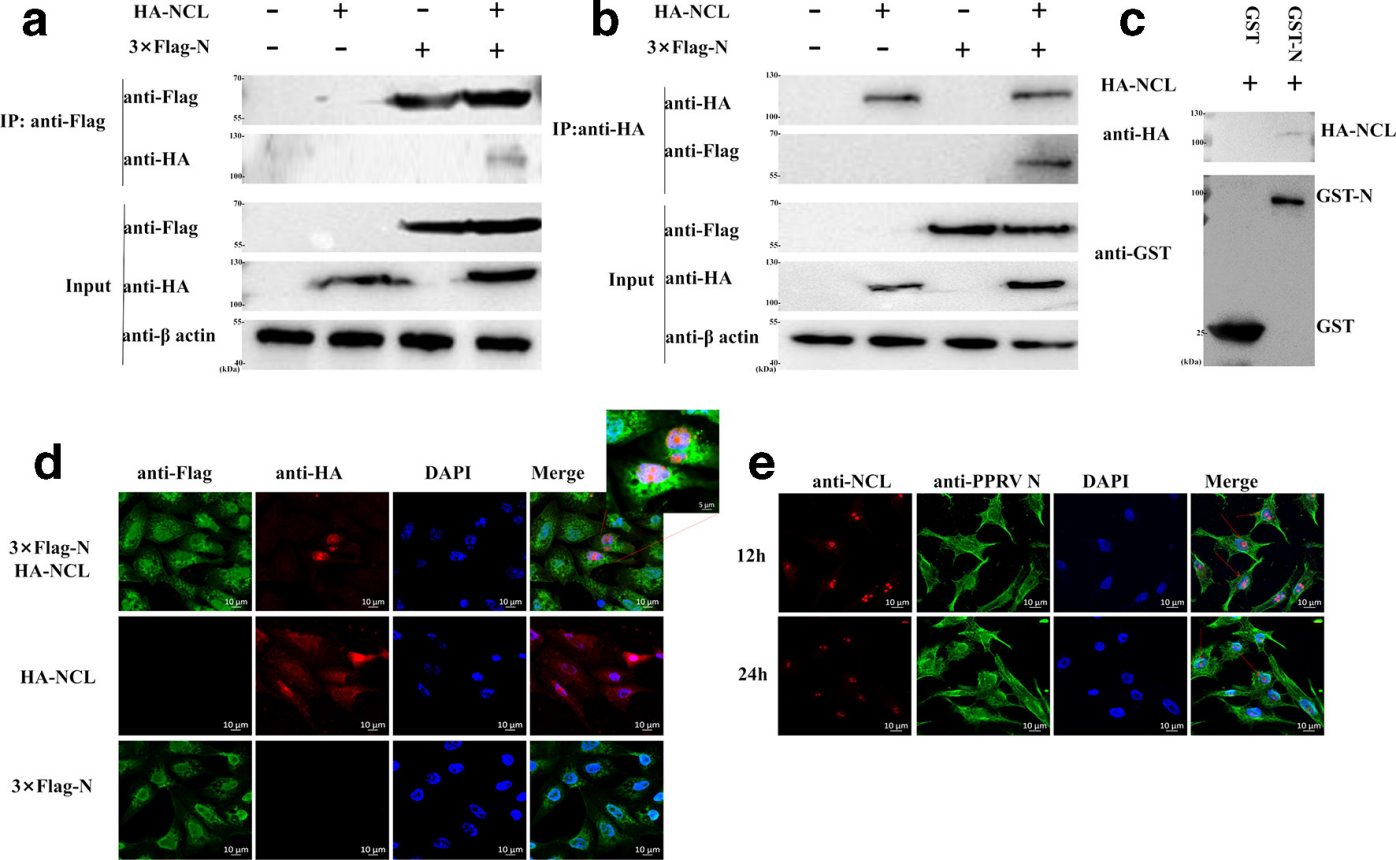
PPRV N protein interacted with NCL. (a, b) Co-IP of N protein with NCL. HEK293T cells were transfected with pHA-NCL and p3 ×Flag-N, individually or in combination. The obtained cell samples were co-immunoprecipitated with anti-Flag MAb (a) or anti-HA MAb (b) and were detected by Western blotting with anti-HA MAb (a) or anti-Flag MAb (b) to confirm the presence of NCL and N protein. (c) GST pulldown assay of N protein and NCL. Glutathione beads conjugated to GST or GST–N protein were incubated with lysates of HEK293T cells transfected with pHA-NCL. Eluted proteins were subjected to SDS-PAGE and NCL was detected by immunoblotting with mouse anti-HA MAb. GST and GST–N protein expression were verified by immunoblotting with mouse anti-GST MAb. (d) Colocalization of N protein with NCL. HEK 293 T cells in 12-well plates (Corning) were cotransfected with pHA-NCL and p3 ×Flag-N. Cells were fixed at 36 h post-transfection and stained with mouse anti-HB MAb and rabbit anti-Flag MAb, followed by incubation with Alexa Fluor 594 goat anti-mouse IgG(H+L) (red) and Alexa Fluor 488 goat anti-rabbit IgG(H+L) (green). The nuclei were stained with DAPI. (e) Colocalization of N protein with NCL in cells infected with PPRV.

To further verify the specific interaction between NCL and N protein, we performed co-IP experiments. In short, HEK293T cells were transfected with pHA-NCL and p3 ×Flag-N, individually or in combination. After 48 h transfection, cell lysates were collected and immunoprecipitated with anti-Flag or anti-HA MAb. The results of Western blotting (Fig. 2a, b) showed that co-IP only occurred when two plasmids were cotransfected, indicating that NCL specifically interacts with N protein in mammalian cells. Since the above experiments cannot fully prove the direct interaction between NCL and N protein, a GST pulldown experiment was performed using purified N protein expressed in *E.coli* and glutathione beads conjugated with GST–N or GST alone. The NCL–N protein interaction was confirmed again in a GST pulldown assay. NCL was pulled down by GST–N protein, but not by GST alone (Fig. 2c).

### Co-localization of NCL and N protein was detected in cells

To examine the co-localization of NCL and N protein in cells, an immunofluorescence assay (IFA) was performed in Vero-SLAM cells by cotransfecting with pHA-NCL and p3 ×Flag-N. As shown in Fig. 2d, both NCL and N protein were distributed in the nucleus and cytoplasm of cells, but NCL was co-localized with N protein in the Vero-SLAM cell nucleus. In addition, we also analysed the co-localization of PPRV N protein and NCL in Vero-SLAM cells naturally infected with PPRV. Our results still showed that NCL and PPRV N protein were co-localized in the nucleus (Fig. 2e).

### NCL specifically binds to the core domain of PPRV N protein (N1) through its C terminus

**Fig. 3.**
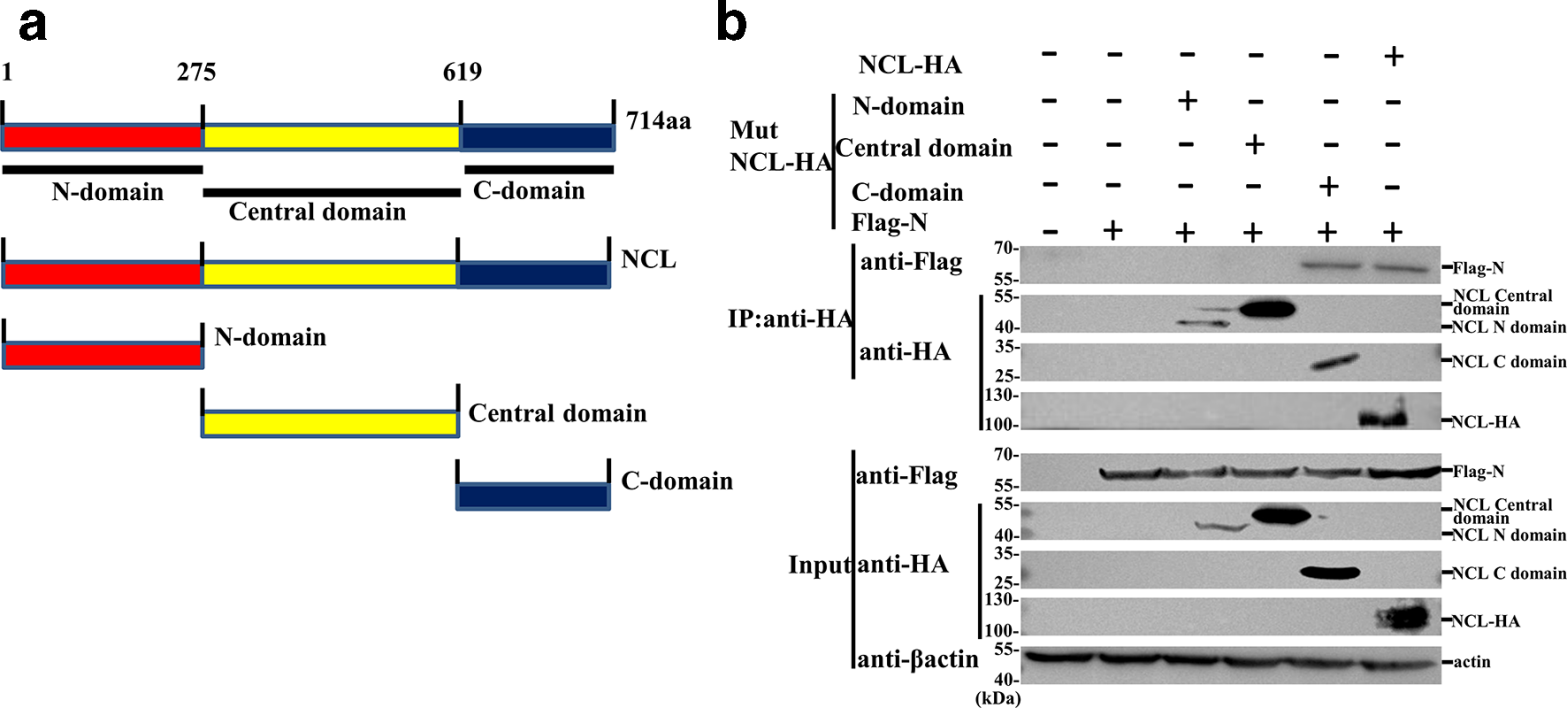
The C-terminal of NCL was necessary for its interaction with PPRV N protein. (a) Schematic representation of the strategy for constructing truncated mutants. The numbers represent amino acid positions. (b) Co-IP analysis of the association of Flag-N protein with HA-tagged NCL or mutant forms of NCL in HEK293T cells co-transfected with indicated plasmids.

To determine the domain of NCL that mediates its binding with N protein, three NCL deletion mutants [pHA-NCL N-domain (1–275), pHA-NCL central domain (276–619) and pHA-NCL C-domain (620–714)] were generated, and then their interactions with N protein were examined by co-IP. The results of co-IP assays proved that the NCL C-domain is responsible for the interaction between NCL and N protein (Fig. 3).

**Fig. 4.**
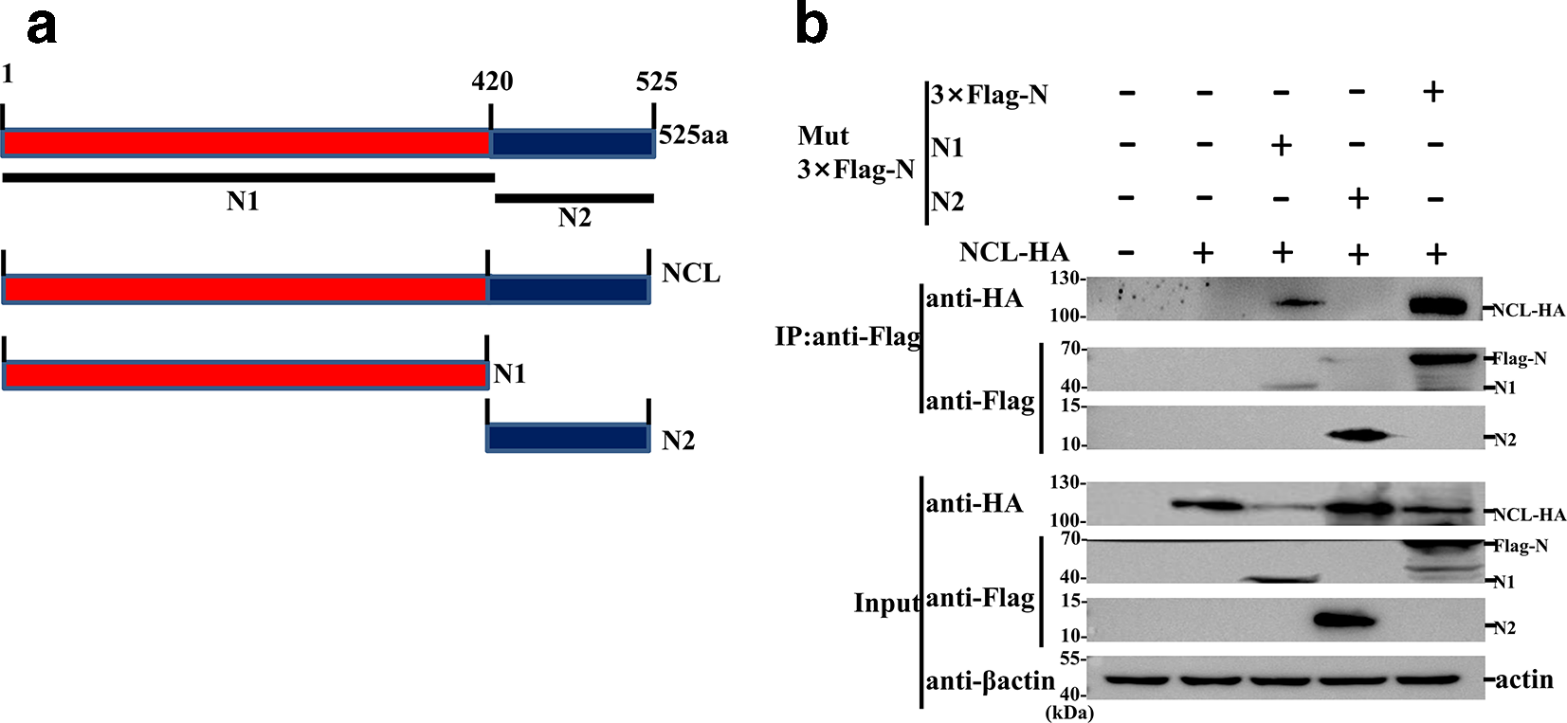
NCL interacted with the core domain of PPRV N protein. (a) Schematic representation of the strategy for constructing truncated mutants. The numbers represent amino acid positions. (b) Co-IP analysis of the association of HA-NCL protein with Flag-tagged N or mutant forms of N in HEK293T cells co-transfected with indicated plasmids.

Using similar strategies, we also identified the domain of N protein that is responsible for the interaction between NCL and N protein. Briefly, two truncated PPRV N protein constructs [p3 ×Flag–N N1(1–420) and p3 ×Flag–N N2 (420–525)] were constructed, and then co-IP assays were performed to examine their interaction with NCL. The results showed that the core domain of PPRV N protein was indispensable for N protein to interact with NCL (Fig. 4).

### Reducing the level of NCL in cells via siRNAs promotes PPRV growth

**Fig. 5.**
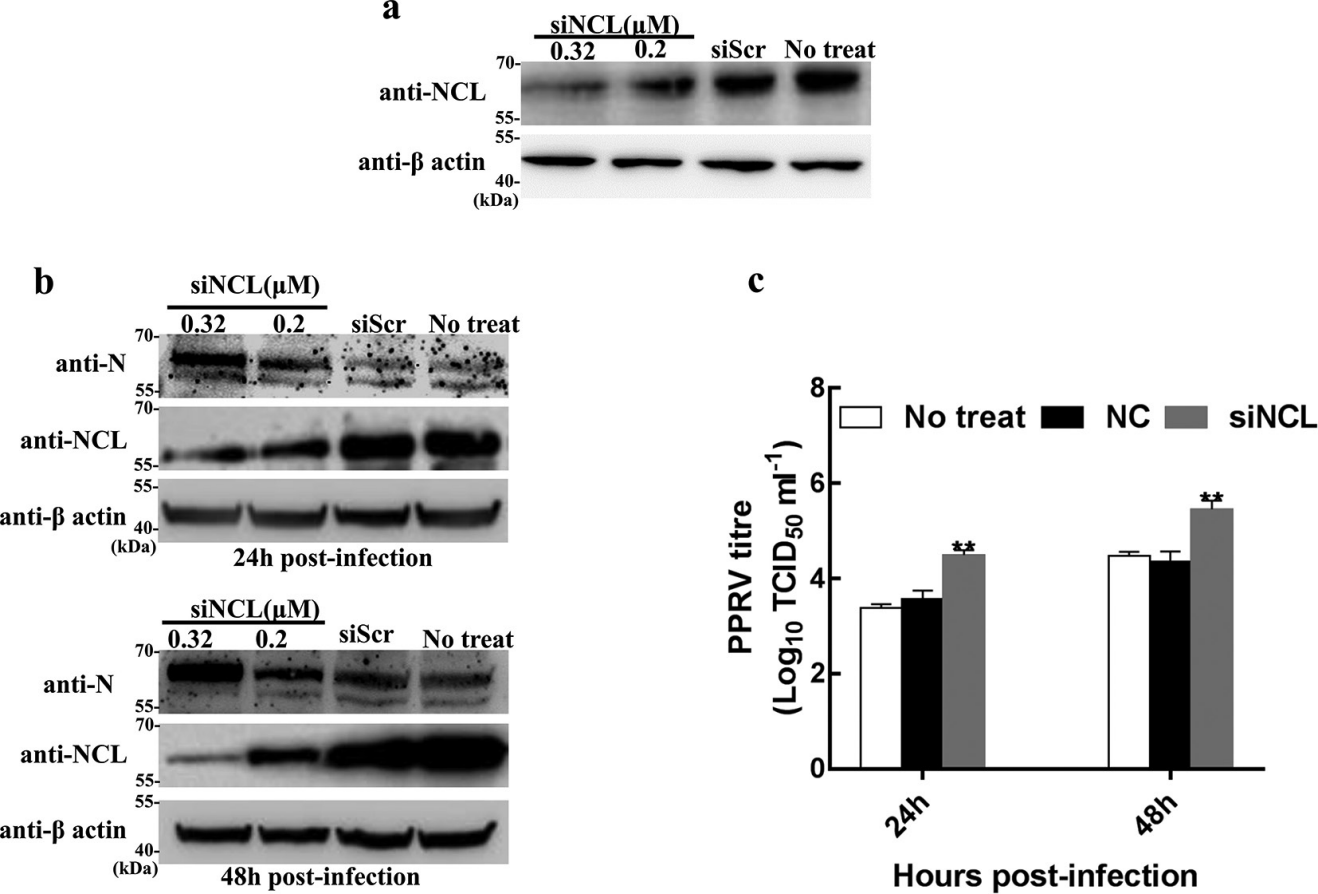
Knockdown of NCL increased growth of PPRV. (a) Detection of NCL expression levels by Western blot assay. Vero-SLAM cells transfected with siNCL, siScr were subjected to immunoblotting with NCL antibody, and untreated cells (no treat) were used as a control. (b) The expression of N protein increased in Vero-SLAM cells treated with siNCL. After transfection with siNCL, siScr for 24 h, cells were infected with PPRV at an m.o.i. of 3. Expression of PPRV N protein and endogenous NCL was detected at 24 h and 48 h after infection by Western blot assay. (c) PPRV titres in NCL-silenced cells. Cells transfected with siRNA were infected with PPRV as described above. Supernatants were collected at 24 h and 48 h after infection for virus titre assay. The error bars represent the standard deviation of the mean for three independent experiments.

To explore the role of NCL in the life cycle of PPRV, RNA interference technology was used to reduce the level of NCL in cells, and then the effect of NCL silencing on PPRV growth was determined. Briefly, Western blotting assay results showed that the expression level of NCL was significantly reduced in Vero-SLAM cells treated with specific siRNAs (Fig. 5a). Subsequently, Vero-SLAM cells treated with siRNA targeting NCL and scrambled siRNA (siScr) were infected with PPRV. The expression level of N protein in all cell samples were analysed by Western blotting, and the titre of PPRV in the infected Vero-SLAM cells was evaluated using the Reed–Muench method. The results showed that silencing the expression of NCL could lead to an increase in N protein (Fig. 5b) and the virus titre of PPRV (Fig. 5c). These results indicated that NCL negatively regulated PPRV growth.

### Overexpression of NCL inhibits PPRV growth

**Fig. 6.**
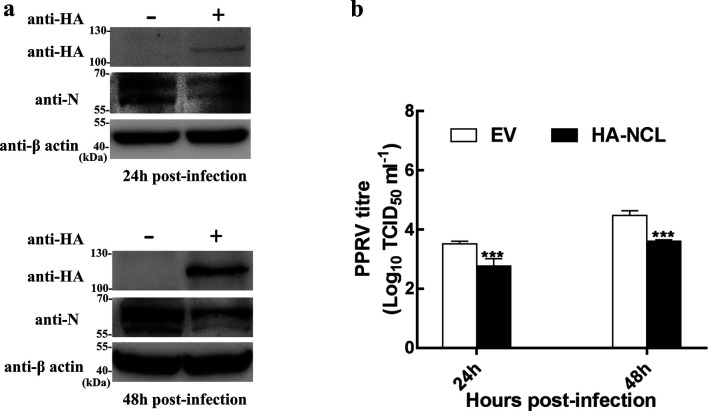
Overexpression of NCL suppressed growth of PPRV. (a) Expression of N was decreased in NCL-overexpressed cells. Vero-SLAM cells were infected with PPRV after transfection of pHA-NCL or empty vector. Cells were harvested for a Western blot assay to analyse the expression of N protein at 24 h and 48 h after infection. (b) PPRV growth was inhibited when NCL was overexpressed. Cells were transfected with specific plasmids and then infected with PPRV as described above. The viral titres of the supernatants collected at 24 h and 48 h post-infection were determined by the Reed–Muench method.

After 24 h transient transfection of the pHA-NCL, Vero-SLAM cells were infected with PPRV, and cell samples were collected at 24 h and 48 h post-infection, respectively. Then the effect of overexpression of NCL on PPRV growth was evaluated by detecting the expression level of N protein and the titre of PPRV. We observed an decrease of N protein (Fig. 6a) and PPRV titre when NCL was overexpressed in Vero-SLAM cells (Fig. 6b), which is in contrast to the results for silencing the expression of NCL in cells.

### NCL inhibits PPRV genomic replication

**Fig. 7.**
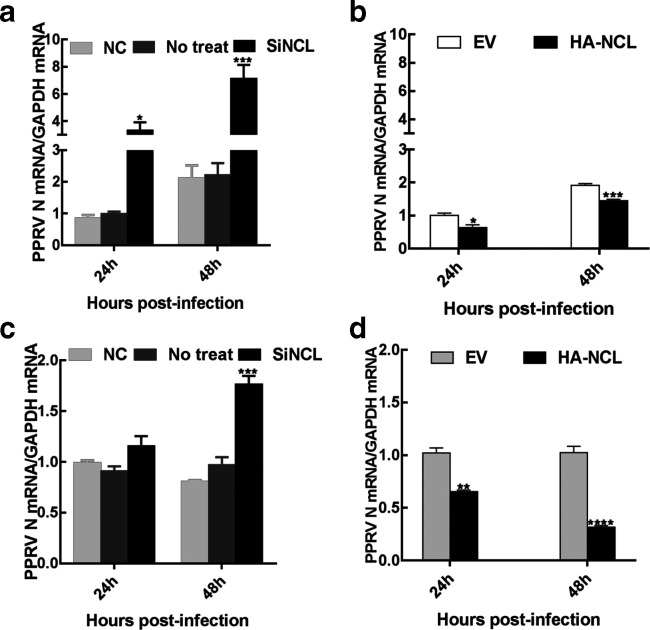
NCL inhibits replication of PPRV genomes. (a,b) Real-time qPCR of NCL-silenced cells infected with PPRV in Vero-SLAM cells after 24 h and 48 h. (c,d) Real-time qPCR of NCL-silenced cells infected with PPRV in EECs after 24 h and 48 h. EV, empty vector.

To investigate the role of NCL in PPRV RNA synthesis, Vero-SLAM cells were transfected with siNCL or siScr and then infected with PPRV. Real-time quantitative (q)PCR confirmed that the number of PPRV genomic RNA copies was increased in NCL knockdown cells but not in cells treated with siScr (Fig. 7a). Conversely, overexpression of NCL resulted in the decline of PPRV genomic replication (Fig. 7b). Further, we repeated this experiment in EECs and a consistent result was obtained (Fig. 7c, d). Taken together, these results showed that NCL inhibited the replication of PPRV genomic.

### NCL stimulates IFN-β activation

**Fig. 8.**
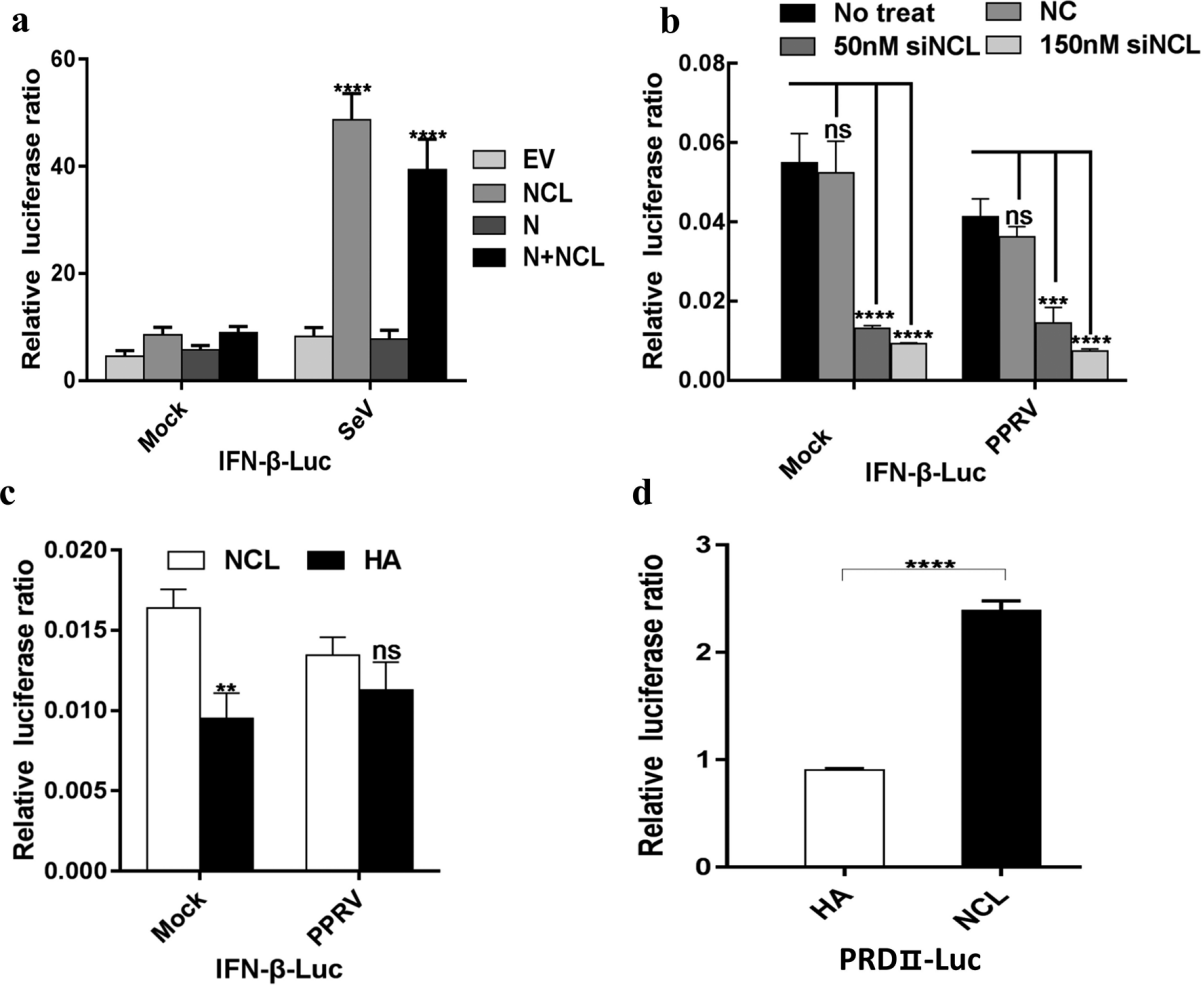
NCL induced IFN-β production by the NF-κB pathway. (a) Expression of the IFN-β in HEK293T cells transfected with the pHA-NCL and/or p3 ×Flag -N or empty vector. NCL increased IFN-β activation in SeV-induced HEK293T cells. (b,c) Expression of the IFN-β in EECs transfected with siNCL and pHA-NCL. (d) Expression of the PRDII in EECs transfected with siNCL and pHA-NCL.

IFN is considered to be a key component of the innate immune response and the first line of defence against viral infection [39]. Hence we analysed whether NCL affects the production of interferon by dual-luciferase reporter assays. When NCL was overexpressed in HEK293T cells, the transcription level of IFN-β was significantly increased compared with the cells transfected with empty vector, indicating that NCL stimulates IFN-β activation (Fig. 8a), and similar results were also observed in EECs. Further studies showed that NCL induced IFN-β production by the NF–κB pathway (Fig. 8b, c and d). Moreover, we found that PPRV infection can block the production of IFN-β, which is consistent with other studies [40].

## Discussion

N protein is one of the important structural proteins of paramyxoviruses such as PPRV, MV and NDV. It has been proved that N protein plays many key roles in the life cycle of PPRV; for example, N protein can promote viral assembly and the formation of genomic RNA capsid. More importantly, N protein is involved in the formation of replication complexes and is responsible for virus replication. Therefore, some host proteins or cytokines inhibit the replication of paramyxovirus by interacting with the N protein. For example, the C-terminal 24 amino acids of the MV nucleocapsid protein represent a regulatory domain that contains a functional motif that mediates direct interaction with Hsp72 [18]. Moreover, the MV nucleocapsid protein (MV-N) specifically binds to a membrane protein receptor that is highly expressed on the surface of thymic epithelial cells. This binding triggers sustained calcium influx and inhibits spontaneous cell proliferation by arresting cells in the G(0) and G(1) phases of the cell cycle [17]. Besides, the viral mRNAs are synthesized from the promoter region at the beginning of N gene, so it would be a good strategy to inhibit morbillivirus replication by silencing N expression. Previous studies have shown that siRNAs targeting PPRV and RPV N mRNA inhibit the replication of virus in infected cells [12]. Subsequently, the motif of the morbillivirus nucleoprotein with the RRWYYDRNUGGUUYGRG (R is A/G, W is A/U, Y is C/U, D is G/A/U and N is any base) was susceptible to RNA interference, the silencing of which leads to the inhibition of N transcript in PPRV, rinderpest virus (RPV)and measles virus (MV). For PPRV, this translated motif is RINWFEN and is located at position 143–149 in the N protein, and among strains of the ﻿family *Paramyxovirus* the central part (NWF) is conserved. Those studies also showed that inhibition of N transcript with subsequent inhibition of M transcript resulted from indirect inhibition of viral RNA-dependent RNA polymerase and transcription complex activity (composed of N, P and L proteins) [9].

In this study, we found that NCL, an important host protein that plays multiple roles in the cell life cycle [24], could interact with PPRV N protein. An increasing number of studies are showing that nucleolins are involved in host regulation of infection and the replication of pathogenic micro-organisms, especially in the development of some tumours. For example, the tumour-necrosis-factor-α-inducing protein (Tipα) of *Helicobacter pylori* binds to the cell-surface NCL in human gastric cancer cell lines, and then the binding complex of NCL and Tipα is internalized into cells, which triggers the deterioration of gastric cancer. Studies have shown that a high level of NCL is positively associated with different grades, extent of invasion and overall survival rate of hepatocellular carcinoma [41]. AS1411 (NCL-targeting DNA aptamer) underwent phase II clinical trials as an anti-metastatic renal cell carcinoma agent [42]. Recent studies have revealed the important role of nucleolins in regulating some viral infection. For example, NCL expressed on the cell surface is a receptor for HIV-1, and combination with midkine leads to inhibition of HIV infection [43]. NCL also acts as a receptor for EV71 [35] and respiratory syncytial virus [44], and mediates the internalization of human parainfluenza virus type 3 into host cells [45]. Besides, NCL interacts with the 3′ untranslated regions of feline calicivirus and is indispensable for virus replication [46]. In addition, NCL plays an important role in Epstein–Barr virus episome maintenance and transcription [47]. These studies are all examples of how nucleolins can positively promote virus growth, and some studies have shown that nucleolins can inhibit virus growth. For example, NCL is identified as a potential antiviral host factor that inhibits influenza A virus (IAV) replication [38]. In our study, we also demonstrated that NCL not only interacts with PPRV N protein, but also plays a negative regulatory role in PPRV growth. As the mechanisms of NCL inhibit PPRV growth, our results indicated that NCL can indirectly inhibit PPRV growth by stimulating the interferon (IFN) pathway (Fig. 8a, b and c). In a word, our study reported for the first time that NCL is an important host restriction factor against PPRV growth, which could provide new clues for understanding the pathogenesis of morbilliviruses.
